# Health Status And Delays In Referral-To-Treatment Waiting Times for Elective Admissions of Adults From Deprived Areas And Ethnic Minority Backgrounds: A Study of Healthcare Inequalities

**DOI:** 10.1007/s40615-025-02515-5

**Published:** 2025-06-18

**Authors:** David Fluck, Emma Jackson, Ana Llamas-Montoya, Amandeep S. Chana, Kevin Kelly, Gareth I. Jones, Jacqui N. Rees, Julia Tudose, Yvonne Jones, Jo S. Finch, Jennifer Davison, Julian Ruse, Chris Jones, Jonathan Robin, Christopher H. Fry, Thang S. Han

**Affiliations:** 1 Medical Director’s Office, Cardiff and Vale University Health Board, Heath Park, Cardiff, CF14 4XW UK; 2 Department of Operations – Restoration and Improvement, Ashford and St Peter’s Hospitals NHS Foundation Trust, Guildford Road, Chertsey, Surrey KT16 0PZ UK; 3 Surrey Heartlands ICB, Surrey Heartlands, Duke’s Court Duke Street, Woking, GU21 5BH UK; 4 Department of Equality, Ashford and St Peter’s Hospitals NHS Foundation Trust, Diversity & InclusionGuildford Road, Chertsey, Surrey KT16 0PZ UK; 5 Department of Information Technology, Ashford and St Peter’s Hospitals NHS Foundation Trust, Guildford Road, Chertsey, Surrey KT16 0PZ UK; 6 Department of Improvement Partnership Team, Ashford and St Peter’s Hospitals NHS Foundation Trust, Guildford Road, Chertsey, Surrey KT16 0PZ UK; 7 Department of Quality, Ashford and St Peter’s Hospitals NHS Foundation Trust, Guildford Road, Chertsey, Surrey KT16 0PZ UK; 8 Department of Performance & Operations, Ashford and St Peter’s Hospitals NHS Foundation Trust, Guildford Road, Chertsey, Surrey KT16 0PZ UK; 9 Department of Medicine, Ashford and St Peter’s Hospitals NHS Foundation Trust, Guildford Road, Chertsey, Surrey KT16 0PZ UK; 10 School of Physiology, Pharmacology and Neuroscience, University of Bristol, Bristol, UK; 11 Department of Endocrinology, Ashford and St Peter’s Hospitals NHS Foundation Trust, Guildford Road, Chertsey, Surrey KT16 0PZ UK; 12 Institute of Cardiovascular Research, Royal Holloway, University of London, Egham, Surrey TW20 0EX UK

**Keywords:** Poverty, Healthcare services, Public health

## Abstract

We studied all patients (total 53,611) whose referral-to-treatment (RTT) ended in hospital admission (“*admitted pathways*”) between 01/2019 and 12/2023. Their health status and RTT waiting times were related to indices of deprivation (IMD: combined data from income, employment, education, health, crime, barriers to housing and services, and living environment) and to ethnicities (87% Caucasians; 6% South Asians; 5.1% Other Ethnicities; 0.9% Blacks; 0.7% Mixed Race; 0.3% Chinese). The risk of RTT waiting times > 18 weeks was assessed by logistic regression, adjusted for confounding factors (age, sex and number of comorbidities) and presented as odds ratios (OR) and 95% confidence intervals: least-deprived areas (IMD decile = 10) and Caucasians were reference levels. Patients’ median age (66 years, interquartile range = 51–77) was representative of “admitted pathways” UK patients. Major chronic diseases were more common amongst patients from ethnic minority and deprived backgrounds. Risks of RTT waiting times > 18 weeks for elective admissions rose progressively with increasing deprivation: OR = 1.20 (1.05–1.36), compared to the least deprived areas; and mixed race: OR = 1.39 (1.10–1.76), other ethnicities: OR = 1.15 (1.05–1.26), and all ethnic minorities: OR = 1.11 (1.05–1.18), compared to Caucasians. Our findings provide evidence for health and healthcare inequalities associated with individuals from deprived areas and ethnic minority backgrounds, particularly those of mixed race. More research and resources are needed to address these inequalities that include policy interventions to reduce barriers that hinder access to healthcare and advocating for policies that address underlying social determinants of health that reduce inequalities in education, employment and housing.

**Supplementary Information:**

The online version contains supplementary material available at https://doi.org/10.1007/s40615-025-02515-5.

## Introduction

In high-income countries such as the UK, poverty and ethnic minority groups are known to be associated with poor health and early deaths [1–3]. Despite national efforts carried out for many years to improve the health of such groups, health gaps between prosperity and poverty and between ethnicities continue to widen [3–5]. In addition to drivers such as access to material resources and health-related behaviours, there is evidence that the needs of disadvantaged groups are often neglected and even worsened by responses from health services [1, 2, 6]. These attempts have been hindered by multiple factors faced by patients as well as healthcare providers, since it is difficult to identify specific factors and challenging to address them [1, 5, 6]. Specifically, inadequate access to healthcare services is thought to be a major barrier for individuals from disadvantaged backgrounds [7–9], but more high-quality empirical data are needed to support healthcare providers in addressing such barriers locally.

The consultant-led referral-to-treatment (RTT) waiting time—a measure of the duration from referral to the first elective (non-urgent) treatment, is a key performance indicator used to monitor performance and effectiveness of a referral programme. Long RTT waiting times suggest delays in access to healthcare services [8], which are detrimental to patients because of inadequate treatment to alleviate pain, disability and anxiety for a prolonged period. Furthermore, their condition may deteriorate, or they may even die whilst waiting [10–13].

National data for RTT waiting times have been published monthly since 2007 for two pathways: “*admitted pathways*” patients whose RTT pathways ended in admission for treatment, and for “*non-admitted pathways*” patients whose RTT pathways ended for reasons other than admission [14]. The National Health Service (NHS) sets a standard that 92% of RTT waiting times should be within 18 weeks. However, this standard has not been met since September 2015 [14]. In view of their poorer health, individuals from deprived areas and ethnic minority groups may also experience longer RTT waiting times. However, little is known about this relationship since nationally published reports on RTT waiting times are based exclusively on data from the entire population, irrespective of their socio-demographic and ethnic minority backgrounds [15].

In this study of a large cohort of elective admissions in “*admitted pathways*” patients, we examined the underlying health status and RTT waiting times of patients living in a wide range of areas, according to the index of multiple deprivation (IMD) and to ethnic minority backgrounds.

## Methods

### Study Design, Participants and Setting

In this cross-sectional study, data of all elective admissions of adults in “*admitted pathways*” were collected between 1 st January 2019 and 31 st December 2023. The study was based in a single NHS hospital, which is the largest provider of hospital services in Surrey, UK, serving a population of 430,000. The median age of 66 years (interquartile range = 51–77) in this study is representative of “*admitted pathways*” patients in the UK [16].

### Selection Criteria

All “*admitted pathways*” patients aged ≥ 18 years were included. “*Non-admitted pathways*” patients were not selected as RTT waiting times do not apply to this group. Patients from obstetrics and mental health were also not included in the present study because their data are separately collected by their own specialties.

### Data Handling

Clinical data were recorded by healthcare professionals and documented through national databases including the personal demographics service and local healthcare records, which are shared securely amongst healthcare providers.

### Sociodemographic and Medical History

Data were recorded for age, sex, history of chronic morbidities, ethnicity and area of residence, as well as history of morbidities including myocardial infarction, cerebrovascular disease, connective tissue disease, congestive heart failure, dementia, diabetes mellitus, liver disease, peptic ulcer disease, peripheral vascular disease, pulmonary disease, non-metastatic and metastatic cancers, paraplegia and renal disease. Information on disease severity, socio-economic status or access to care was not available. Ethnicities were self-identified by patients according to the list defined by the National Health Service: Caucasian, South Asian, Black, Chinese, mixed race, and other ethnicities. RTT waiting times (in days) for elective admissions were assessed from the date of referral to the date of admission for treatment. Areas of deprivation were indicated by IMD (see below).

### Index of Multiple Deprivation

The IMD was established by the Ministry of Housing, Communities and Local Government [17], which ranked each of 32,844 small areas in England from most to least deprived based on a combination of seven domains of deprivation including “*Income Deprivation*” (measures a situation where a significant portion of a population in a specific area experiences low income, encompassing both those out of work and those in work but with low earning) and “*Employment Deprivation*” (measures the proportion of the working-age population involuntarily excluded from the labour market. This includes people who would like to work but are unable to do so due to unemployment, sickness or disability or caring responsibilities); “*Education, Skills and Training Deprivation*” measures the lack of attainment and skills in the local population; “*Health Deprivation and Disability*” measures the risk of premature death and the impairment of quality of life through poor physical or mental health; “*Crime*” measures the rate of recorded crime for four major volume crime types—burglary, theft, criminal damage and violence—representing the risk of personal and material victimisation at a small area level; “*Barriers To Housing And Services*” measures the physical and financial accessibility of housing and local services. The indicators fall into two sub-domains: geographical barriers, which relate to the physical proximity of local services, and wider barriers, which include issues relating to access to housing such as affordability; and “*Living Environment*” measures the quality of the local environment. The indicators fall into two sub-domains. The indoors living environment measures the quality of housing, while the outdoors living environment contains measures of air quality and road traffic accidents. The derived IMD scale is divided into ten equal groups (deciles) with the lowest 10% (decile 1) being the most deprived area and the highest 10% (decile 10) the least deprived area [17]. Thus, an IMD assigned to an individual is based on their area of residence.

### Categorisation of Data

IMD was categorised into each decile except for deciles 2 and 3 which were combined due to the low number of patients in decile 2 (there were no patients in IMD decile 1). The threshold for breach of RTT waiting times was set according to a guideline at 18 weeks. Multimorbidity was defined as ≥ 2 conditions [18].

### Statistical Analysis

All analyses were performed using IBM SPSS Statistics, v28.0 (IBM Corp., Armonk, NY). Chi-squared tests were used to examine categorical data including differences between RTT waiting times > 18 weeks and ethnic groups or IMD categories. Kruskal–Wallis tests were used to assess differences between multiple groups for non-normally distributed continuous data. Logistic regression analysis was conducted to assess the risk of RTT waiting times > 18 weeks from deprivation or ethnic minority and presented in two models: unadjusted and adjusted for potential confounding factors including age, sex and number of comorbidities. Interaction terms were added to these models to test for potential interaction between independent variables. The least deprived areas (IMD decile 10) and Caucasians were used as references for comparison between deprivation status and between ethnic groups, respectively.

## Results

### Characteristics

A total of 53,611 adults (53.2% women) of median age 66 years (interquartile range: 51–77) were studied. The numbers of patients rose progressively from the youngest age group (18–29 years), peaking at the second oldest age group (70–79 years), followed by a decrease in the oldest (≥ 80 years). There were no patients from the most deprived areas (IMD decile 1), and only a few from the second most deprived areas (IMD decile 2). By contrast, almost half of all patients (48%) were from the three least deprived areas (IMD deciles 8–10). Most patients were Caucasian (87.0%), followed by South Asian (6.0%), other ethnicities (5.1%), Black (0.9%), Mixed Race (0.7%) and Chinese (0.3%), i.e. a total of 13.0% from ethnic minority backgrounds. The RTT waiting times were achieved by 77.9%, i.e. 22.1% breached the target. Most patients had no morbidities (57.1%), the remainder had one morbidity (30.3%) or ≥ 2 morbidities (12.7%) (Table 1).

**Table 1:** Characteristics of 53,611 adult patients referred for elective surgical and medical admissions between 1^st^ January 2019 and 31^st^ December 2023

	*n*	%
Age (years)		
18–39	6992	13.0
40–59	13,314	24.8
60–79	23,429	43.7
≥ 80	9876	18.4
Sex		
Men	25,116	46.8
Women	28,495	53.2
Index of multiple deprivation*		
2	123	0.2
3	1416	2.6
4	4911	9.2
5	4895	9.1
6	4926	9.2
7	6230	11.6
8	7374	13.8
9	9850	18.4
10	8412	15.7
Ethnicity		
White (Caucasian)	46,652	87.0
South Asian	3192	6.0
Black	488	0.9
Chinese	181	0.3
Mixed race	351	0.7
Other (non-white)	2747	5.1
All ethnic minorities	6959	13.0
Number of comorbidities		
None	30,612	57.1
1 comorbidity	16,227	30.3
2 comorbidities	5026	9.4
4 comorbidities	1746	3.3
RTT waiting times		
≤ 18 weeks	41,741	77.9
> 18 weeks	11,870	22.1

*Index of multiple deprivation information available for 48,137 patients, i.e. 5474 (10.2%) cases with missing values

### Ethnicity and Deprivation

In the most deprived areas (IMD deciles 2–4), 81.9–82.1% of patients were Caucasians, which rose progressively to 90–91% in the least deprived areas (IDM deciles 8–10). Conversely, 8.4–9.9% were represented by South Asians and 1.5–1.8% by Blacks in the most deprived areas, which decreased progressively to 3.1–4.0% and 0.5–0.6% respectively, in the least deprived areas. A similar pattern was observed for mixed race and other ethnicity groups, whilst Chinese followed the pattern for Caucasians. Overall, 17.9–18.1% were represented by all ethnic minorities in the most deprived areas and 9.2–9.7% in the least deprived areas (Table 2).

**Table 2:** Distribution of individuals from different ethnic groups according to index of multiple deprivation scores

	Index of multiple deprivation scores								
	2–3	4	5	6	7	8	9	10	Total
Ethnicity	**%**	**%**	**%**	**%**	**%**	**%**	**%**	**%**	**%**
Caucasian	82.1	81.9	85.3	89.9	89.5	90.3	90.6	90.8	88.7
South Asian	8.4	9.9	6.5	3.5	3.9	4.0	3.8	3.1	4.7
Black	1.8	1.5	1.1	0.8	0.6	0.5	0.5	0.6	0.8
Chinese	0.2	0.5	0.3	0.3	0.2	0.3	0.4	0.4	0.3
Mixed race	0.7	0.8	0.5	0.6	0.8	0.4	0.6	0.4	0.6
Other ethnicities	6.8	5.3	6.2	4.9	5.0	4.4	4.1	4.8	4.9
All ethnic minorities	17.9	18.1	14.7	10.1	10.5	9.7	9.4	9.2	11.3

### Distribution of Patients Admitted to Specialties

Almost half of all elective admissions were referred to the busiest three specialties: ophthalmology (20.2%), trauma and orthopaedics (16.9%) and colorectal surgery (11.6%). The remaining 51.3% of patients were referred to other specialities (Supplementary Table 1).

### Health Status

Thirteen of the most common chronic diseases were recorded in this study. Myocardial infarction, diabetes, liver disease, peptic ulcer disease, pulmonary disease and non-metastatic cancer emerged most commonly in those from the most deprived areas, which progressively become less common in those from the least deprived areas. Conversely, non-metastatic cancers were more common in the least deprived areas, but the pattern was less apparent for metastatic cancers.

Liver disease was highest in the most deprived areas (IMD 2 and 3), whilst pulmonary disease and non-metastatic cancers continue to be more common in Caucasians. There were proportionally more South Asians with a history of myocardial infarction, diabetes and peptic ulcer disease, whilst there were more Caucasians with a history of pulmonary disease and non-metastatic cancers, but there were no ethnic differences in metastatic cancers (Supplementary Table 2).

Within each age decade, diabetes (Supplementary Fig. 1A) and multi-morbidities (≥ 2 conditions) (Supplementary Fig. 1B) were proportionally more common in individuals from the more deprived areas compared to those from the less deprived areas from the age of 40 years. Diabetes was more common amongst South Asians and Blacks from the age of 50 years (Supplementary Fig. 1C). Multi-morbidities were most common in Asians from 70 years, and in Blacks in the 70–79 age group (Supplementary Fig. 1D).

### RTT Waiting Times According to Ethnic and IMD Groups

**Fig. 1:**
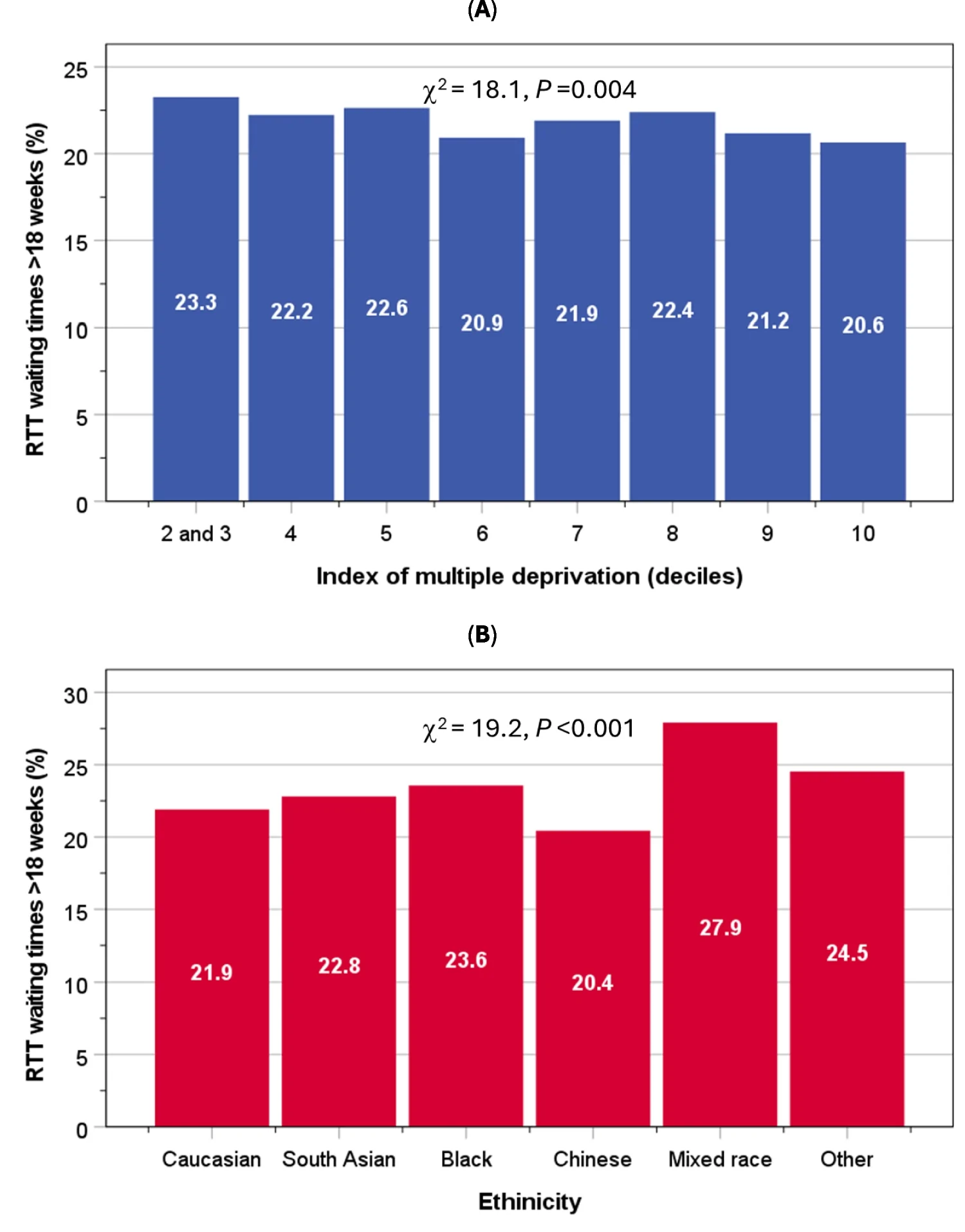
Rates of RTT waiting times > 18 weeks for elective admissions according to the index of multiple deprivation (**A**) and ethnicity (**B**)

The rates of RTT waiting times > 18 weeks were highest amongst those from the most deprived areas (IMD deciles 2 and 3; 23.3%), whilst those from the least deprived areas were smaller (IMD decile 10; 20.6%) (Fig. 1A). Caucasians (21.9%) and Chinese (20.2%) had the lowest rates of RTT waiting times > 18 weeks, whilst South Asians (22.8%), Blacks (23.6%), mixed race (27.9%) and other ethnic minority groups (24.5%) had higher rates (Fig. 1B).

**Fig. 2:**
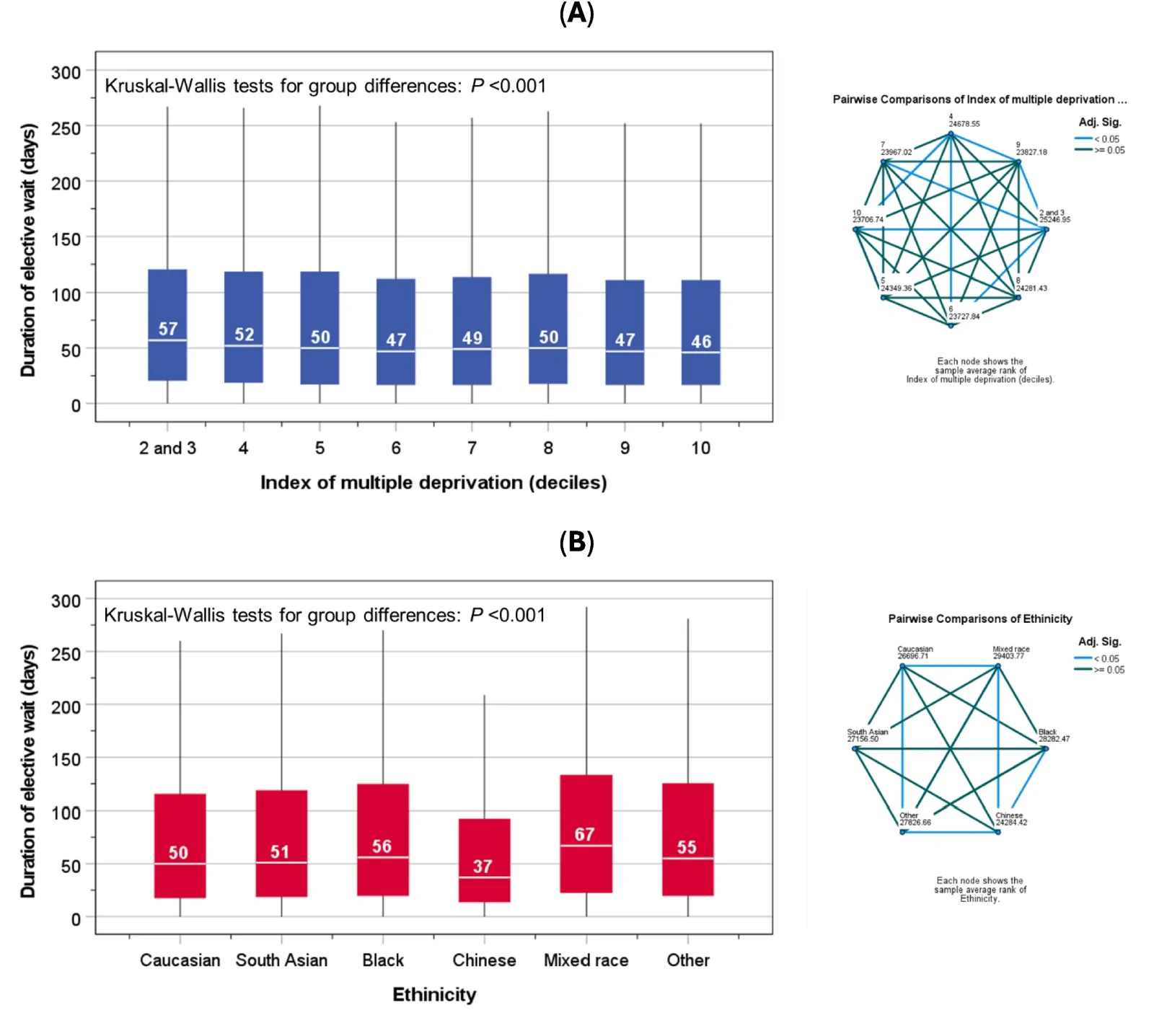
Length of RTT waiting times for elective admissions according to the index of multiple deprivation (**A**) and ethnicity (**B**). Note: boxes indicate median and interquartile range values and whiskers indicate 95% intervals

The median RTT waiting times were longest for patients from the most deprived areas (IMD deciles 2 and 3) at 57 days, which were progressively reduced to 46 and 47 days for those from the least deprived areas (IMD deciles 10 and 9) (Fig. 2A). The median RTT times were shortest for Chinese (37 days) and Caucasian (50 days) groups, whilst patients from Black (56 days) and mixed race ethnicity (67 days) groups had the longest times (Fig. 2B).

### Risk of Having an RTT Waiting Time > 18 Weeks

Compared to individuals from the least deprived areas (IMD decile 10), the adjusted risks for RTT waiting times > 18 weeks for elective admissions rose progressively with increasing deprivation, with an odds ratio for those from the most deprived areas of 1.20 (1.05–1.36). There was also an increased risk of RTT waiting times > 18 weeks for the all ethnic minorities group, odds ratio = 1.11 (1.05–1.18); the mixed race group, odds ratio = 1.39 (1.10–1.76): and the other ethnicities group, odds ratio = 1.15 (1.05–1.26), compared to Caucasians. There was no significant risk of RTT > 18 weeks for South Asians, Blacks and Chinese compared to Caucasians (Table 3). There was no evidence of interaction between ethnicity and IMD with any other factors in the prediction of RTT.

**Table 3:** Logistic regression analysis to estimate the risk of RTT waiting times > 18 weeks in ethnic minorities and in patients from deprived areas

		Risk of RTT waiting times for elective admission > 18 weeks					
		Unadjusted			Adjusted for age, sex, and number of morbidities		
	Rates (%)	OR	95% CI	*P*	OR	95% CI	*P*
IMD deciles							
10 (reference)	20.6	1	–	–	1	–	–
9	21.2	1.03	0.96–1.11	0.381	1.04	0.97–1.12	0.300
8	22.4	1.11	1.03–1.20	**0.007**	1.12	1.04–1.21	**0.003**
7	21.9	1.08	1.00–1.17	0.065	1.09	1.01–1.18	0.031
6	20.9	1.02	0.93–1.11	0.700	1.03	0.95–1.13	0.454
5	22.6	1.12	1.03–1.22	**0.007**	1.15	1.06–1.25	**0.001**
4	22.2	1.10	1.01–1.20	**0.031**	1.12	1.03–1.22	**0.011**
3 and 2	23.3	1.16	1.02–1.33	**0.021**	1.20	1.05–1.36	**0.007**
Ethnicity							
Caucasians (reference)	21.9	1	–	–	1	–	–
Mixed race	27.9	1.38	1.09–1.75	**0.007**	1.39	1.10–1.76	**0.006**
South Asians	22.8	1.05	0.97–1.15	0.232	1.06	0.98–1.16	0.158
Blacks	23.6	1.10	0.89–1.36	0.377	1.10	0.89–1.36	0.368
Chinese	20.4	0.92	0.64–1.32	0.635	0.88	0.61–1.26	0.480
Other ethnicities	24.5	1.16	1.06–1.27	**0.001**	1.15	1.05–1.26	**0.002**
All ethnic minorities	23.7	1.10	1.05–1.18	** < 0.001**	1.11	1.05–1.18	** < 0.001**

*RTT* referral to treatment, *IMD* index of multiple deprivation

## Discussion

### Key Findings

In this study of over 50,000 patients, we observe that compared to individuals from less deprived areas and Caucasians, those from more deprived areas and from ethnic minority backgrounds including South Asian, Black and those of mixed race had worse underlying health and delays in access to healthcare services.

### Health Status

Our observations of poorer health amongst individuals from the most deprived areas or from ethnic minority backgrounds, compared to those from less deprived areas or Caucasians respectively, support findings from previous reports [1, 3, 15]. However, the important feature of our study includes a wide range of health conditions (13 in total) which enabled us to examine in-depth how diseases presented differently amongst individuals from residential areas of different IMD as well as those of different ethnicities. Because there was a partial overlap between ethnic minority and deprivation, many diseases were more prevalent in these two components, whilst there was also a discord in presentation for certain diseases. These findings suggest that exposure to risk factors may differ between ethnic groups for a given degree of deprivation (see below).

### Mechanisms Driving Health Disparities

The prevalence of diabetes was highest in patients from the most deprived areas and ethnic minorities, emerging from an early age (40 years) and is consistent with those from previous studies [19, 20]. There remain many arguments regarding the reason for an increase in diabetes amongst individuals from more deprived neighbourhoods. It has been suggested that poor lifestyle due to an inability to afford healthy food and lack of physical activity due to a lack of safe areas for exercise were some of the main reasons [21, 22]. Renal disease was also common amongst patients from more deprived areas and ethnic minorities. We observed that renal disease was more common in those with diabetes compared to those without: 11.4% vs. 4.6% (*P* < 0.001), suggesting that renal disease is a complication of diabetes in many patients.

Pulmonary disease was also highly prevalent amongst patients from more deprived areas. Poor housing, overcrowding, poor heating [23] and environmental pollution [24] may be contributing factors. In contrast to diabetes, pulmonary disease was more common in Caucasians. Studies have shown that amongst Caucasians smoking was more common [25] and four times more started at a young age (15 years) [26] than other ethnic groups and that smoking and related chronic obstructive pulmonary disease is more common in low annual income households [27].

Liver disease was not common compared to other conditions in this study. The observation of an increased prevalence amongst the middle-aged group (40–59 years) in the most deprived areas is consistent with findings of a higher mortality amongst individuals < 75 years old from the most deprived areas, compared to those from least deprived areas (30.3 vs. 14.9 deaths per 100,000 population) [28]. Several possible causes have been suggested, including the use of hepatotoxic substances such as excessive alcohol consumption and a greater risk of exposure to infectious diseases [29].

There was a contrasting association between IMD and cancer—non-metastatic cancers were more common whilst metastatic cancers were less common amongst individuals in high IMD categories (less deprived areas). This may indicate better awareness and cancer screening amongst those in more prosperous areas, whilst those from poorer areas lack these opportunities and diagnosis of cancers may be delayed, leading to a higher risk of metastatic cancers [29]. These patterns were similarly observed in ethnic groups for non-metastatic cancers, which were more common in Caucasians. However, there were no ethnic differences for metastatic cancers. Studies have shown that this may be due to reduced access to health care, screening and treatment amongst those from deprived backgrounds [29, 30]. Better access without delay minimises the risk through early detection and disease progression [5].

### Delays in RTT Waiting Times

Few studies have examined waiting times in those from deprived neighbourhoods or ethnic minorities [31, 32]. Previous studies have suggested that people with low income tend to neglect their health behaviour to a greater extent, which could affect waiting times [33–35] although ethnic minorities were more commonly from more deprived neighbourhoods than Caucasians. The relationship between RTT waiting times and ethnic minorities is complex as there may be a number of factors, apart from poverty, contributing to their differences. Discrimination (conscious or unconscious bias) may also play a role [36]. To avoid long RTT waiting times, more people, often those who can afford the costs, are increasingly seeking private health care [37].

Although it is generally accepted that RTT waiting times are affected by missed appointments, termed “did not attend” (DNA) [38, 39], there is a lack of quantitative data to support this association. Data from our hospital showed DNA rates over a period of 3 months (April–June 2023) were 8.7% for patients from the most deprived areas (IMD deciles 2 and 3) compared to 4.9–5.3% for those from the least deprived areas (IMD deciles 9 and 10). This compares with DNA rates of 8.5–9.0% amongst Black or Asian (Pakistani) patients, 7% amongst mixed race, compared to 4.8% in Chinese and 5.7–6.9% in Caucasians. These differences in DNA rates between prosperity and poverty and between ethnic backgrounds mirror those observed for RTT, lending support to the suggestion that DNAs lead to longer RTT waiting times.

A mixed race profile constitutes one of the most rapidly growing ethnic populations in the UK and US [40]. However, information on their health is scanty [40, 41]. This may in part, be due to the complexity of categorisation for this group [42]. Our findings of the mixed race group having the longest RTT waiting times are novel and provide valuable information for assessing their health outcomes. The underlying reasons for this observation remain unclear and cannot be deduced entirely from their DNA rates or deprivation background—both of which were worse than those of Caucasians, but not too different from those of single ethnic minority groups (Blacks or Asians).

There is a paucity of data on direct comparison between mixed race and single ethnic minority groups. Existing published data have mainly focussed on health disparities between those of mixed race and Caucasians. These differences therefore can only help to explain longer RTT waiting times in general, but not against single ethnic minority groups as observed in this study. Previous studies have found that those from a mixed race background faced racial microaggressions in health care similar to that in daily settings [43] and which had an impact on their mental health. A study of high school students showed that those from a mixed race background had higher rates of poor mental health and fewer protective factors compared to Caucasians [44]. A previous study has suggested that adults perceive their multiracial (mixed race) identity to influence mental health more than physical health [45]. Another study observed that a higher proportion of multiracial participants reported feeling that “the healthcare provider thought they were not smart, the provider acted superior, and not being listened to by the provider than other groups”. Those who experienced healthcare discrimination were more than twice as likely to report poor health status [46].

In general, there is a lack of knowledge on the health of individuals from a mixed race background, compared to that in single ethnic groups. More research is necessary to identify aetiological factors that may have a bearing on their well-being and engagement with healthcare systems. These include culture, household, and family structure (such as being a single parent, safeguarding, and adoption status), socioeconomic status and educational level.

### Mechanisms Driving Disparities in RTT Waiting Times and DNA Rates

The mechanisms driving disparities in RTT waiting times and DNA rates are factors mostly outside of patients’ control that have a more profound impact on ethnic minority groups. These include a lack of resources relating to transport and cost issues and difficulty in taking time off work [47], mental health issues which may deter them from attending appointments [48] and communication such as a language barrier which may cause confusion to patients concerning their appointments, as well as difficulty in cancelling or rearranging appointments. It is recognised that there are other factors that could affect a patient’s attendance, including those who often change their contact and those who experience insecure or supported accommodation [38].

Guidelines have been advocated by the NHS England to help reduce DNA rates relating specifically to health inequalities [49]. It is recommended that systems and providers should take steps to ensure they are not exacerbating health inequalities in relation to their approach to managing DNAs. These include completing an equality and health inequalities impact assessment for outpatient services and engagement with patients including those with lower health literacy to facilitate access appointments easily. This approach adopted by the North Lincolnshire Clinical Commissioning Group in England led to a 90% reduction of DNA rates [50]. This included options available for patients’ preferred means of communication, appointment times and appointment types (e.g. remote or fact-to-face consultation) and assessment of the organisation’s local levels of digital literacy to allow those who do not have access to a mobile telephone to receive and amend appointments.

### Limitations and Strengths

In this study, we chose “admitted pathways” patients rather than “non-admitted pathways” patients on the basis that delays in RTT waiting times would have a greater impact on their health. Nevertheless, studies on “non-admitted pathways” patients are being conducted in a separate study. Data for obstetrics and mental health were not included in the present study, but it would be of interest to perform similar research in these groups in the future which could provide additional insights into healthcare inequalities. Except for information on the stage of cancer—non-metastatic or metastatic (see above), this study did not have information on the disease severity of other diseases diagnosis. It is possible that individuals from ethnic minority or deprived groups may suffer from more severe conditions, given their longer RTT waiting times. It would be important to compare our findings with other healthcare systems from other countries in Europe and the US where healthcare is funded differently. Furthermore, prospective studies exploring disease progression while awaiting treatment could yield valuable real-world evidence on the impact of delayed access. The 10.2% of patients with missing IMD data had similar clinical characteristics to those who had complete data; therefore this is unlikely to introduce bias to the results observed in this study. These missing values did not affect the results for the relationship between ethnicities and outcomes. We used the UK definition of an 18-week threshold to indicate delay in access to health care, but the definition of delay varies between countries [29, 31], so that caution should be taken when conducting international comparisons. Under-representation of more deprived areas and ethnic minorities could limit the generalisability of the findings. The strengths of this study lie in its large number of patients with a comprehensive clinical profile, and its design that allowed us to compare RTT waiting times between a wide range of IMD and ethnicities. Furthermore, the IMD used in this study is a well-established measure of deprivation and is widely used by the National Health Service. In addition to assessing individual ethnic groups (Caucasian, South Asian, Black, Chinese, mixed race, and other ethnicities), we also combined all ethnic minorities together for comparison with Caucasians. This helped to overcome the limitation of a relatively small number of patients from ethnic minority backgrounds. This type of categorisation is sometimes used in healthcare analysis, but we do recognise that different ethnic groups vary in health outcomes and lived experiences [51]. To reduce such health inequalities, it will be important to conduct further research to determine the underlying reasons at the individual level for both higher DNA rates and longer RTT waiting times amongst patients from deprived backgrounds or ethnic minorities.

## Conclusions

Our findings provide evidence for long-standing health and healthcare inequalities associated with individuals from deprived areas and of ethnic minority backgrounds, particularly those from mixed race groups. More research and resources are needed to address these inequalities to improve the health of these individuals. These include policy interventions with a focus on reducing barriers to accessing healthcare and advocating for policies that address the underlying social determinants of health by strengthening community-based initiatives to reduce inequities in education, employment and housing.

## Supplementary Information

Below is the link to the electronic supplementary material.

Supplementary file1 (DOCX 269 KB)

## Data Availability

Data not available.
