# Supplementary material for: Health Status And Delays In Referral-To-Treatment Waiting Times for Elective Admissions of Adults From Deprived Areas And Ethnic Minority Backgrounds: A Study of Healthcare Inequalities
Source: J Racial Ethn Health Disparities. 2025 Jun 18;13(5):3544–55. doi: 10.1007/s40615-025-02515-5 (PMC13546329; doi:10.1007/s40615-025-02515-5)
Supplement: Supplementary file 1 [file 40615_2025_2515_MOESM1_ESM.docx]

**Supplementary Table 1.** Distribution of patients according to admitted specialties.

| **Treatment code** | **Description** | **n** | **%** |
| --- | --- | --- | --- |
| 100 | General Surgery | 608 | 1.13 |
| 101 | Urology Service | 3046 | 5.68 |
| 103 | Breast Surgery | 1918 | 3.58 |
| 104 | Colorectal Surgery | 6238 | 11.64 |
| 106 | Upper Gastrointestinal Surgery Service | 1274 | 2.38 |
| 107 | Vascular Surgery Service | 1346 | 2.51 |
| 109 | Bariatric Surgery Service | 124 | 0.23 |
| 110 | Trauma and Orthopaedic | 9071 | 16.92 |
| 120 | Ear Nose and Throat Service | 1870 | 3.49 |
| 130 | Ophthalmology | 10808 | 20.16 |
| 140 | Oral Surgery | 264 | 0.49 |
| 143 | Orthodontic Service | 14 | 0.03 |
| 144 | Maxillofacial Surgery Service | 3549 | 6.62 |
| 145 | Oral and Maxillofacial Surgery | 97 | 0.18 |
| 160 | Plastic Surgery | 40 | 0.07 |
| 170 | Cardiothoracic Surgery | 7 | 0.01 |
| 180 | Emergency Medicine Service | 17 | 0.03 |
| 190 | Anaesthetic | 2056 | 3.84 |
| 191 | Pain Management | 2747 | 5.12 |
| 300 | General Internal Medicine | 100 | 0.19 |
| 301 | Gastroenterology | 4142 | 7.73 |
| 302 | Endocrinology Service | 23 | 0.04 |
| 303 | Clinical Haematology Service | 1599 | 2.98 |
| 306 | Hepatology | 144 | 0.27 |
| 307 | Diabetes | 107 | 0.20 |
| 310 | Audio Vestibular Medicine | 2 | 0.00 |
| 315 | Palliative Medicine | 11 | 0.02 |
| 320 | Cardiology | 1689 | 3.15 |
| 324 | Anticoagulant Service | 5 | 0.01 |
| 327 | Cardiac Rehabilitation | 6 | 0.01 |
| 328 | Stroke Medicine | 21 | 0.04 |
| 329 | Transient Ischaemic Attack | 3 | 0.01 |
| 330 | Dermatology | 109 | 0.20 |
| 340 | Respiratory Medicine | 235 | 0.44 |
| 361 | Renal Medicine | 33 | 0.06 |
| 400 | Neurology Service | 68 | 0.13 |
| 410 | Rheumatology | 58 | 0.11 |
| 430 | Elderly Medicine | 45 | 0.08 |
| 450 | Dental Medicine | 40 | 0.07 |
| 812 | Diagnostic Imaging | 1 | 0.00 |
| 840 | Audiology Service | 76 | 0.14 |
|  | **Total** | **53611** | **100** |

**Supplementary Table 2.** Comorbidities amongst patients from different areas of Index of multiple deprivation and ethnic background in older adults.

|  | **Index of multiple deprivation** | | | | | | | | |
| --- | --- | --- | --- | --- | --- | --- | --- | --- | --- |
|  | **2 and 3**  **(n=1,539)** | **4**  **(n=4,911)** | **5**  **(n=4,895)** | **6**  **(n=4,926)** | **7**  **(n=6,230)** | **8**  **(n=7,374)** | **9**  **(n=9,850)** | **10**  **(n=8,412)** | **Linear trend** |
| **Conditions** | **%** | **%** | **%** | **%** | **%** | **%** | **%** | **%** | ***P**** |
| Myocardial infarction | 5.2 | 5.1 | 5.3 | 4.2 | 5.0 | 4.9 | 4.6 | 3.8 | **<0.001** |
| Cerebrovascular accident | 0.4 | 0.5 | 0.7 | 0.8 | 0.7 | 0.8 | 0.8 | 0.6 | 0.148 |
| Connective tissue disorders | 2.7 | 3.1 | 3.3 | 2.7 | 3.1 | 3.1 | 3.6 | 2.9 | 0.305 |
| Congestive heart disease | 1.7 | 2.1 | 2.8 | 2.6 | 2.7 | 2.7 | 2.3 | 2.6 | 0.335 |
| Dementia | 0.6 | 0.7 | 0.8 | 0.9 | 0.9 | 1.0 | 1.1 | 1.0 | **0.006** |
| Diabetes | 15.9 | 14.7 | 14.4 | 12.3 | 11.3 | 11.8 | 11.0 | 10.0 | **<0.001** |
| Liver disease | 1.0 | 0.6 | 0.8 | 0.7 | 0.7 | 0.6 | 0.7 | 0.4 | **0.013** |
| Peptic ulcer disease | 1.2 | 0.9 | 0.9 | 0.8 | 0.6 | 0.7 | 0.7 | 0.7 | **0.050** |
| Peripheral arterial disease | 1.0 | 1.0 | 1.0 | 1.3 | 1.3 | 0.9 | 0.9 | 0.9 | 0.063 |
| Pulmonary disease | 19.7 | 17.9 | 17.4 | 17.8 | 16.7 | 15.2 | 14.3 | 13.8 | **<0.001** |
| Non-metastatic cancer | 8.1 | 9.5 | 11.0 | 12.5 | 12.5 | 13.4 | 13.4 | 14.5 | **<0.001** |
| Metastatic cancer | 1.4 | 2.4 | 2.0 | 2.0 | 2.9 | 2.2 | 2.3 | 1.9 | 0.864 |
| Paraplegia | 0.2 | 0.4 | 0.4 | 0.5 | 0.4 | 0.3 | 0.3 | 0.4 | 0.312 |
| Renal disease | 6.0 | 4.8 | 5.5 | 6.1 | 5.1 | 6.2 | 5.3 | 5.2 | 0.840 |
| Renal disease | 6.0 | 4.8 | 5.5 | 6.1 | 5.1 | 6.2 | 5.3 | 5.2 | 0.840 |
|  | **Ethnicity** | | | | | | | | |
|  | **Caucasian**  **(n=4,6652)** | **Asian**  **(n=3,192)** | **Black**  **(n=488)** | **Chinese**  **(n=181)** | **Mixed race (n=351)** | **Other ethnicities (n=2,747)** | **Linear trend** | **All minorities**  **(n=6,959)** | **χ^2^ test*** |
| **Conditions** | **%** | **%** | **%** | **%** | **%** | **%** | ***P*** | **%** | ***P**** |
| Myocardial infarction | 4.6 | 5.7 | 2.0 | 0.6 | 4.0 | 3.6 | **0.031** | 4.4 | 0.262 |
| Cerebrovascular accident | 0.7 | 0.5 | -- | -- | 0.9 | 0.4 | **0.006** | 0.4 | **0.004** |
| Connective tissue disorders | 3.2 | 2.2 | 4.3 | 3.3 | 5.4 | 2.3 | **0.004** | 2.6 | **0.004** |
| Congestive heart disease | 2.5 | 1.6 | 1.2 | 0.6 | 0.9 | 1.7 | **<0.001** | 1.6 | **<0.001** |
| Dementia | 0.9 | 0.4 | 0.4 | -- | 0.9 | 0.7 | **0.015** | 0.6 | **0.001** |
| Diabetes | 11.3 | 22.7 | 15.8 | 8.8 | 15.7 | 15.9 | **<0.001** | 18.1 | **<0.001** |
| Liver disease | 0.6 | 0.6 | 0.2 | -- | 0.9 | 0.7 | 0.614 | 0.6 | 0.285 |
| Peptic ulcer disease | 0.7 | 0.6 | 0.8 | 1.1 | -- | 1.1 | **0.037** | 0.8 | 0.172 |
| Peripheral arterial disease | 1.0 | 0.4 | -- | 0.6 | 0.3 | 0.7 | **<0.001** | 0.5 | **<0.001** |
| Pulmonary disease | 16.2 | 13.7 | 13.7 | 7.7 | 16.2 | 11.1 | **<0.001** | 12.6 | **<0.001** |
| Non-metastatic cancer | 12.8 | 6.0 | 7.0 | 5.5 | 4.0 | 6.2 | **<0.001** | 6.0 | **<0.001** |
| Metastatic cancer | 2.2 | 2.2 | 2.0 | 5.5 | 1.1 | 1.7 | 0.211 | 2.0 | 0.134 |
| Paraplegia | 0.4 | 0.3 | 0.2 | 0.6 | -- | 0.2 | 0.189 | 0.3 | 0.122 |
| Renal disease | 5.5 | 4.8 | 5.7 | 2.8 | 4.8 | 4.7 | **0.010** | 4.8 | **0.004** |

*Differences between Caucasian and all minorities.

**Supplementary Figure 1.** Age-adjusted (decades) rates of diabetes (**A**), and multiple morbidities according to the index of multiple deprivation (**B**), and diabetes and multiple morbidities according to ethnicities (**C and D**).

**Figure 1.**

(A) **(B)**

(**C**)	(**D**)
